# Serum Lipid Levels and Risk Of Hand Osteoarthritis: The Chingford Prospective Cohort Study

**DOI:** 10.1038/s41598-017-03317-4

**Published:** 2017-06-09

**Authors:** M. Garcia-Gil, C. Reyes, R. Ramos, M. T. Sanchez-Santos, D. Prieto-Alhambra, T. D. Spector, D. J. Hart, N. K. Arden

**Affiliations:** 1 Research Unit, Family Medicine. Institut Universitari d’Investigació en Atenció Primària Jordi Gol (IDIAP Jordi Gol), Girona, Spain; 2 Translab Research Group. Department of Medical Sciences, School of Medicine, University of Girona, Girona, Spain; 3 GREMPAL Research Group, Institut Universitari d’Investigació en Atenció Primària Jordi Gol [IDIAP Jordi Gol], Barcelona, Spain; 4 CIBERFes, Universitat Autònoma de Barcelona and Instituto de Salud Carlos III, Barcelona, Spain; 5 Primary Care Services, Catalan Institute of Health (ICS), Girona, Spain; 6 Nuffield Department of Orthopaedics, Rheumatology and Musculoskeletal Sciences, University of Oxford, Oxford, United Kingdom; 7 Arthritis Research UK Sports Exercise and Osteoarthritis Centre of Excellence, University of Oxford, Oxford, United Kingdom; 8 MRC Lifecourse Epidemiology Unit, University of Southampton, Oxford, United Kingdom; 9 Department of Twin Research & Genetic Epidemiology, King’s College London, Oxford, United Kingdom

## Abstract

The development of hand osteoarthritis (HOA) could be linked to hyperlipidaemia. No longitudinal studies have addressed the relationship between serum lipid profile and HOA. The study aim was to determine the association between serum lipid profile and the incidence of radiographic hand osteoarthritis (RHOA). All women in a prospective population-based cohort from the Chingford study with available baseline lipid measurements and without RHOA on a baseline were included. Study outcome was the incidence of RHOA in year 11 of follow-up. Serum lipid profile variables were analysed as continuous variables and categorised into quartiles. The association between serum lipid profile and RHOA was modeled using multivariable logistic regression. Overall RHOA incidence was 51.6% (45.7–57.4%). An inverse association between HDL cholesterol levels and the incidence of RHOA was observed by quartile: OR of 0.36 [95%CI 0.17–0.75], 0.52 [95%CI 0.26–1.06], and 0.48 [95%CI 0.22–1.03]. Triglycerides levels showed a significant trend. No relationship was found with total or LDL cholesterol. Higher levels of HDL cholesterol appear to protect against RHOA after 11 years of follow-up. More research is needed to elucidate HOA risk factors, the mechanisms related to the lipid pathway, and the effects of lipid-lowering agents on reducing the incidence of OA.

## Introduction

Osteoarthritis (OA) is a prevalent and disabling disease that contributes to increased co-morbidity and is more common in the elderly. The association between OA and cardiovascular disease (CVD) has attracted a large portion of research attention in recent years. These two health conditions frequently co-exist and the disease burden is only partly explained by age. Cardiovascular mortality is increased in individuals with OA^1^ and a high prevalence of CVD risk factors has been described in these individuals^2–4^, suggesting the existence of common pathogenic mechanisms. However, the causal association is not clear^5^.

Substantial evidence has also linked obesity and metabolic syndrome with OA^6–12^. In addition, recent experimental and epidemiological studies suggest that the biological mechanisms involved in the development of OA could be linked to atherosclerosis and the lipid metabolic pathway^13–16^, particularly in non-weight-bearing joints like those of the hand^17–19^. The estimated prevalence of radiological hand osteoarthritis (RHOA) in the elderly is about 80%, and there is evidence of sex-related and genetic predisposition. Risk factors such as age, manual labour, repetitive movement of the hand, overweight, smoking, and age at menopause have been associated with RHOA^20^. However, no longitudinal studies have addressed the relationship between serum lipid profile and OA in non-weight-bearing joints.

Our study aimed to determine if serum lipid profile is associated with incident radiographic hand osteoarthritis (RHOA).

## Methods

### Setting and participants

Participants were selected from the Chingford Women’s Study, a prospective, population-based cohort study that has been described in detail elsewhere^21^. Briefly, all women aged 45–64 years registered at a large general practice in Chingford, North London (UK), were contacted in 1988–1989 and asked to participate in a population-based study to evaluate risk factors for osteoporosis and OA. Of 1,353 women contacted, 1,003 (78%) attended the baseline visit (Y1) and signed their informed consent to participate. All women without RHOA on a baseline hand X-ray were included. The study participants were similar to women in the UK general population in terms of weight, height, and body mass index (BMI)^12^.

### Radiographic assessment of RHOA

A standard hand X-ray was carried out at Y1 and at Y11. At both timepoints, RHOA was scored according to the Kellgren and Lawrence (K & L) scale by a single trained observer (DJH), blinded to clinical details, using the figures and legends of the original atlas^22^. The protocols used for radiographic classification and reproducibility have been previously reported^23, 24^. Briefly, observer kappa statistics for both the interphalangeal and carpo-metacarpal joint groups ranged from 0.7 to 1.0 without any significant differences in the reproducibility of the clinical and radiological measures. RHOA was considered to be present if the women had either proximal or distal interphalangeal joint OA (PIP and DIP, respectively) with a K & L score ≥2 in two or more joints. The outcome was defined as having RHOA at Y11.

### Serum lipid profile measurements

The exposure of interest was the fasting serum lipid profile at the baseline visit (Y1): Total cholesterol (TC), HDL-cholesterol (HDL-c), LDL-cholesterol (LDL-c) and triglycerides (TG) at baseline, following a previously published protocol^11^.

### Study variables and covariates

The following variables were characterized at baseline and included in the analysis: age, any current medication, diabetes medication, statins use, hormone replacement therapy (HRT), previous CVD, menopause, age at menopause, smoking, extent of job-related activity, body mass index (BMI), and systolic and diastolic blood pressure. Age, age at menopause, BMI, and systolic and diastolic blood pressure were treated as continuous variables. Smoking was classified as never smoked, ex-smoker, or currently smoking. Extent of activity on the job was classified as sedentary, half sedentary and half active, and predominantly manual, obesity as BMI ≥ 30, and daily sports activity as none, light (1 hour walking, bowling, golf, badminton, cycling, or swimming), moderate (2 hours of light sport or 1 hour keep-fit or aerobics activity) and intensive (>1 hour keep-fit or aerobics, squash).

### Statistical analysis

Continuous variables were described as mean and standard deviation and categorical variables as percentages. Differences between groups were based on the t-test or chi-square, as appropriate. Cumulative incidence and 95% confidence intervals (CI) for RHOA were also calculated for a 11-year follow-up period.

Logistic regression models were used to evaluate the association between lipid serum profile and incidence of RHOA at Y11. Lipid serum profile was considered as both a continuous and categorical set of variables to evaluate the effect of a potential dose-response relationship with RHOA. Three different models were built to explore this association: Model 1 was non-adjusted, Model 2 was age-adjusted, and Model 3 added the potential confounders that were epidemiologically relevant or statistically significant in the bivariate analysis. Log-rank test was used to contrast the dose-response relationship within serum lipid profile categories.

We used STATA software (version 12) for all the analysis, with two-sided tests. P-values < 0.05 were considered statistically significant.

The study was approved by the Outer North East London Research Ethic Committee and conducted according the rules of good research practices of the Declaration of Helsinki. Each study participant provided written informed consent before participating.

## Results

**Figure 1:**
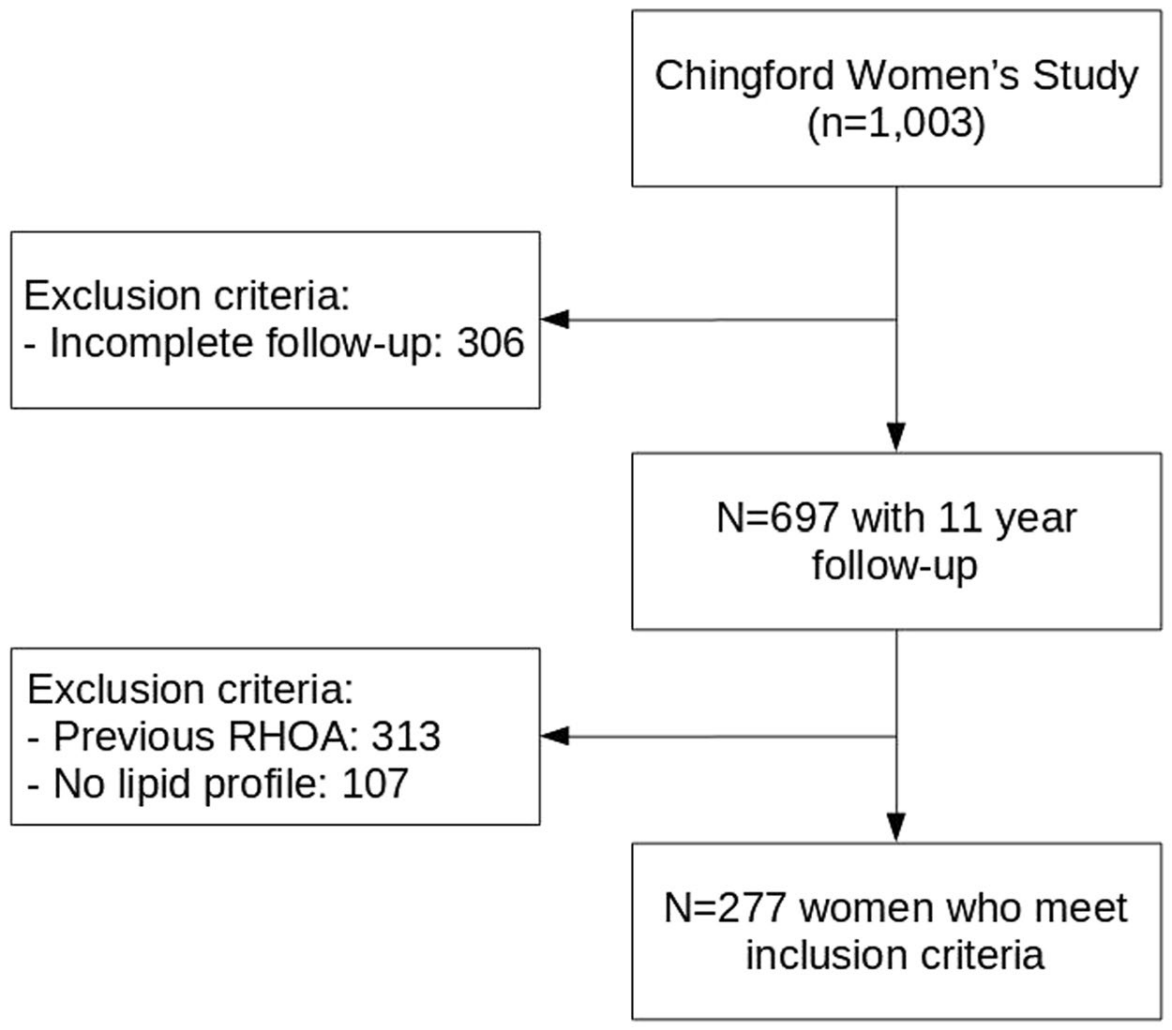
Flowchart of the study population.

Of the 1,003 women participating at baseline, 306 were lost to follow-up (30%) and 697 completed the year 11 follow-up, of which 313 (45%) had RHOA at baseline and 107 (15%) had no baseline lipid serum profile measurements data. The final sample available for analysis was 277 (40%) participants. Baseline participant characteristics are reported in Table 1 and the study flowchart is shown in Fig. 1.

**Table 1:** Baseline characteristics of the study population.

	Final study population N = 277	Lost to follow-up (N = 306)	No serum lipid profile (N = 107)
	N (%)	N (%)	N (%)
Age*	50.4 (4.8)	55.4 (6.1)	54.4 (4.7)
Any current education	81 (29.2)	141 (46.2)	41 (38.2)
Blood pressure medication	30 (10.8)	50 (16.3)	18 (16.8)
Diabetes medication	6 (2.1)	1 (0.3)	—
Statins	8 (2.9)	5 (14.7)	7 (6.5)
Previous CVD	3 (1.0)	18 (5.9)	1 (0.9)
Menopause	163 (58.8)	247 (80.9)	88 (82.2)
Age at menopause*	45.9 (5.7)	47.6 (5.1)	47.2 (4.9)
Previous HRT	70 (25.2)	79 (25.8)	27 (25.2)
Smoking			
Ex-smoker	62 (22.3)	75 (24.5)	29 (27.1)
Current	69 (24.9)	79 (25.8)	18 (16.8)
Extent of activity in job			
Sedentary	11 (4.0)	12 (4.1)	13 (54.8)
Half sedentary + half active	211 (77.0)	236 (78.9)	37 (25.6)
Predominantly manual	52 (19.0)	51 (17.0)	10 (9.6)
Sport			
None	183 (66.7)	220 (73.3)	57 (54.8)
Light	38 (13.8)	39 (13.0)	20 (19.2)
Moderate	35 (12.7)	16 (5.3)	17 (16.3)
Intensive	18 (6.8)	25 (8.4)	10 (9.6)
Obesity	24 (8.6)	58 (18.9)	15 (14.0)
BMI^*^	24.8 (3.9)	26.1 (5.0)	25.5 (3.8)
Blood pressure (mmHg)*			
Systolic	122.4 (19.9)	130.3 (20.1)	128.8 (15.2)
Diastolic	76.4 (11.2)	79.6 (11.5)	78.1 (9.2)
Glucose mmol/L*	4.9 (0.1)	5.0 (0.8)	—
TG mmol/L*	1.1 (0.6)	1.2 (0.6)	—
TC mmol/L*	6.3 (1.3)	6.7 (1.3)	—
HDL-c mmol/L*	1.7 (0.4)	1.7 (0.4)	—
LDL-c mmol/L*	4.1 (1.1)	4.4 (1.2)	—

Numbers are percentages unless otherwise stated.

*Mean (standard deviation).

CVD: cardiovascular disease; HRT: hormone replacement therapy.

TG: triglycerides, TC: total cholesterol, HDL-c: high-density lipoprotein cholesterol, LDL-c: low-density lipoprotein cholesterol.

The final study population was younger and had a healthier cardiovascular profile than women lost to follow-up and those without serum lipid profile measurements, although the percentages of smokers and of women with manual-labour jobs were higher in the final study population, compared to those without lipid profile measurements.

The mean age of the final study population was 50.4 (SD: 4.8) years and BMI was 24.8 (SD: 3.9). About 59% were post-menopausal, 19% had a predominantly manual-labour job, and just 8.6% were classified as obese.

### Association between lipid serum profile and RHOA

Overall cumulative RHOA incidence was 51.6% [45.7–57.4%]. Compared to those without RHOA at Y11, incident RHOA women were older (51.1 [SD: 5.0]), more likely to take blood pressure medication (13.95% vs 7.9%) and had higher mean levels of triglycerides at baseline [1.0 (0.6) vs (1.2 (0.7)], with no other significant differences (Table 2).

**Table 2:** Baseline characteristics of study population according to incident radiographic hand osteoarthritis (RHOA) at 11 years of follow-up.

	No RHOA n = 134	RHOA n = 143	P-value
Age*	49.6 (4.6)	51.1 (5.0)	0.009
Any current medication	36 (26.8)	45 (31.4)	0.40
Blood pressure medication	10 (7.4)	20 (13.9)	0.08
Diabetes medication	4 (2.9)	2 (1.4)	0.36
Statins	4 (2.9)	4 (2.8)	0.92
Previous CVD	2 (1.5)	1 (0.7)	0.52
Menopause	73 (54.4)	90 (62.9)	0.15
Age at menopause*	45.4 (5.8)	46.3 (5.60)	0.31
Previous HRT	29 (21.6)	41 (28.6)	0.18
Smoking			
Ex-smoker	24 (1.9)	38 (26.5)	0.21
Current	34 (25.3)	35 (24.4)	
Extent of activity in job			0.76
Sedentary	7 (5.2)	4 (2.8)	
Half sedentary + half active	102 (76.7)	109 (77.3)	
Predominantly manual	24 (18.1)	28 (19.8)	
Sports activity			0.28
None	93 (69.9)	90 (63.8)	
Light	15 (11.2)	23 (16.3)	
Moderate	19 (14.2)	16 (11.3)	
Intensive	6 (4.5)	12 (8.5)	
Obesity	14 (10.4)	10 (6.9)	0.30
BMI*	24.70 (4.1)	24.95 (3.7)	0.60
Blood pressure (mmHg)			
Systolic	122.0 (20.9)	122.7 (19.9)	0.79
Diastolic	75.8 (11.3)	76.9 (11.1)	0.42
Glucose mmol/L*	4.9 (0.9)	4.9 (0.8)	0.99
TG mmol/L*	1.0 (0.6)	1.2 (0.7)	0.01
TC mmol/L*	6.2 (1.2)	6.4 (1.3)	0.06
HDL-c mmol/L*	1.7 (0.4)	1.7 (0.4)	0.43
LDL-c mmol/L*	3.9 (1.1)	4.1 (1.2)	0.14

Numbers are percentages unless otherwise stated. *Mean (standard deviation)

CVD: cardiovascular disease; HRT: hormone replacement therapy, BMI: body mass index; TG: triglycerides, TC: total cholesterol, HDL-c: high-density lipoprotein cholesterol, LDL-c: low-density lipoprotein cholesterol.

Crude data and age- and fully adjusted logistic regression modelling results are detailed in Table 3. No significant association was found between continuous HDL-c and incident RHOA. HDL-c levels were inversely associated with the incidence of RHOA, with crude OR 0.32 [95%CI 0.16–0.66], 0.50 [95%CI 0.25–0.99], and 0.50 [95%CI 0.23–1.04] for the 2^nd^, 3^rd^, and 4^th^ quartiles compared to the 1^st^ quartile (reference group); this association remained unchanged and showed a significant trend after adjusting for all pre-specified confounders (OR: 0.36 [95%CI 0.17–0.75], 0.52 [95%CI 0.26–1.06], and 0.48 [95%CI 0.22–1.03], respectively).

**Table 3:** Association between lipid measurements and incident radiographic hand osteoarthritis (RHOA) at 10 years of follow-up.

Lipid profile	RHOA		
	Crude OR	Age-adjusted OR	Fully adjusted OR
**HDL-c**			
*Continuous*	0.79 (0.44–1.41)	0.74 (0.41–1.34)	0.76 (0.42–1.39)
*Categorical*			
First quartile (0.72–1.4)	1	1	1
Second quartile (1.5–1.6)	0.32 (0.16–0.66)	0.34 (0.17–0.71)	0.36 (0.17–0.75)
Third quartile (1.7–2.0)	0.50 (0.25–0.99)	0.49 (0.24–0.99)	0.52 (0.26–1.06)
Fourth quartile (2.1–3.4)	0.50 (0.23–1.04)	0.47 (0.22–0.99)	0.48 (0.22–1.03)
**TG**			
*Continuous*	1.68 (1.11–2.54)	1.50 (0.98–2.29)	1.47 (0.93–2.32)
*Categorical*			
First quartile (0.40–0.73)	1	1	1
Second quartile (0.76–1.00)	1.73 (0.92–3.25)	1.54 (0.81–2.93)	1.56 (0.82–2.99)
Third quartile (1.01–1.40)	1.86 (0.95–3.65)	1.59 (0.79–3.19)	1.57 (0.76–3.22)
Fourth quartile (1.42–4.9)	2.28 (1.16–4.46)	1.91 (0.95–3.84)	1.83 (0.87–3.86)
**TC**			
*Continuous*	1.18 (0.98–1.42)	1.10 (0.90–1.34)	1.09 (0.89–1.33)
*Categorical*			
First quartile (3.1–3.6)	1	1	1
Second quartile (5.7–6.4)	1.57 (0.84–2.96)	1.46 (0.77–2.77)	1.43 (0.75–2.71)
Third quartile (6.5–7.4)	1.08 (0.56–2.06)	0.92 (0.47–1.80)	0.91 (0.46–1.80)
Fourth quartile (7.5–11.2)	2.32 (1.15–4.69)	1.81 (0.86–3.81)	1.75 (0.82–3.70)
**LDL-c**			
*Continuous*	1.00 (0.99–1.00)	1.00 (0.99–1.00)	1.00 (0.99–1.00)
*Categorical*			
First quartile (1.41–3.40)	1	1	1
Second quartile (3.42–4.09)	1.33 (0.58–2.18)	1.21 (0.62–2.34)	1.14 (0.58–2.23)
Third quartile (4.15–5.03)	1.62 (0.83–3.18)	1.35 (0.67–2.71)	1.30 (0.64–2.63)
Fourth quartile (5.07–7.80)	1.45 (0.72–2.92)	1.18 (0.57–2.44)	1.15 (0.55–2.39)

*Quartiles (limits in mmol/L).

Models adjusted for age, body mass index, blood pressure medication.

**Trend tests (fully adjusted models): HDL-c, high-density lipoprotein cholesterol (p = 0.009); TG, triglycerides (p = 0.033); TC, total cholesterol (p = 0.278); LDL-c, low-density lipoprotein cholesterol (p = 0.495).

Conversely, TG levels were directly associated with RHOA incidence in the unadjusted continuous logistic model but the association was not significant after adjustment for age. Test for linear trend was significant in the fully adjusted categorical logistic model. No associations were found with TC and LDL-c levels and RHOA over 11 years of follow-up.

## Discussion

In this population-based cohort study, higher levels of HDL-c were found to be associated with a lower incidence of RHOA over 11 years of follow-up. Conversely, the effect size observed in each TG category showed an increased, but not significant, risk for HOA. However, TG levels seem to confer a higher risk of RHOA according to the trend in test results.

Our results disagree with those of a previous cross-sectional study in the Chingford cohort^11^, in which the authors found an association between radiological knee OA and moderately elevated TC but no association between knee OA and higher levels of LDL-c, TG, or HDL-c. In a subanalysis in women with both hand and knee OA, no association was found between any of the components of the serum profile and OA. Our study, based on the same cohort, showed a significant association and a trend in HDL-c because of differences in study design and baseline characteristics of the women included. In the previous study, women with previous RHOA were included, and they were older and had a worse CVD profile, compared to our study participants.

Other studies have shown contradictory results on the relationships between TC, TG, LDL-C and OA incidence. The Rotterdam study^8^, which aimed to analyse the connection between HOA and overweight, detected no association between HOA and TC/HDL-c ratio, whereas our findings that HDL-c levels and TG levels were associated with HOA are consistent with other studies such as those of Davis-Tuck, who found higher levels of TC and TG associated with bone marrow lesions in asymptomatic middle-aged women^25^ and Frey, who showed an association between incident HOA and hyperlipidaemia, particularly at younger ages but could not analyse the effect of serum lipid profile on HOA as it was modified because of statin exposure^26^. Our study population was similar in age and cardiovascular profile to all three of these earlier studies.

Mechanisms through which lipid metabolism could lead to OA still must be fully elucidated. There is evidence that higher HDL-c levels in the synovial fluid have a protective effect^27^, vascular insults affecting the bone marrow next to the cartilage^25^, and the toxic effect of cholesterol at the joint itself ^28^ might contribute to the development of OA. We were able to detect the protective effect of HDL-c and TG on RHOA as a trend in our population, but not an increased risk of RHOA with high TC or LDL-c levels. High HDL-c levels might be present in healthier individuals with better physical status and lower prevalence of comorbid conditions^29^, which could also lead to a lower prevalence and incidence of OA. Women in our study were younger and had normal weight to overweight, better cardiovascular profile, and no previous cardiovascular disease, compared to other studies^7, 11, 17, 18^.

Potential limitations of this study should be mentioned. First, the power of the study to show significant effects might be limited as we only included 40% of the total eligible population, given the combination of high baseline prevalence (45%) of RHOA and the lack of data on cholesterol levels for 15% of the patients. Differences between these 277 participants and the rest of the women included must be considered and could limit the generalizability of our findings. Women included in the study were healthier and younger than those excluded, which might partly explain the lack of association between incident RHOA and certain components of the lipid serum profile, but also might explain the observed association with other components of serum profile, such as TG and HDL-c levels^25, 26^.

Secondly, serum lipid profile was only measured at Y1, and we were not able to determine if the lipid levels detected were previously present or simply reflected an increase at the time of measurement that could be influenced by day-to-day diet and random error. In the same way, changes in lipid serum levels between Y1 and Y11 were not reported, which prevented us from drawing any conclusion regarding the possible impact of serum lipid variations on the outcome or the time lapse between the increase or decrease of these lipids and the outcome. Nonetheless, baseline risk factors may be connected in the overall life-course trajectories.

Thirdly, other unmeasured confounders, such as diet, activity of daily living, or socioeconomic status could have influenced our results.

Finally, we had no information on lipid-lowering drugs taken by our population at either baseline or during follow-up, which could have influenced the nonsignificant association observed between TC and RHOA. We would speculate, however, that the proportion of women treated with such therapies was low at the time of recruitment, as statins had not yet been launched on the market.

## Conclusion

No significant associations between serum lipid profile and RHOA were found after 10 years of follow-up, although significant trends were observed in HDL-c and TG associations. Higher HDL-c levels appear to be protective and higher TG levels seem to confer a higher risk of RHOA.

Treatments available today for RHOA are mainly focused on the symptomatic relief and functional improvement of the joints affected. The burden of OA is bound to increase due to the lack of effective disease-modifying therapies. With this in view, more research is needed to elucidate the aetiology and risk factors of HOA and, specifically, the mechanisms related to the lipid pathway as well as to the potential effects of lipid-lowering agents on the reduction of OA incidence. New pharmacological interventions, especially at early stages, could reduce OA incidence and influence the progression of the disease.
